# Music and the Meeting of Human Minds

**DOI:** 10.3389/fpsyg.2018.00762

**Published:** 2018-05-16

**Authors:** Alan R. Harvey

**Affiliations:** School of Human Sciences, The University of Western Australia, Perron Institute for Neurological and Translational Science, Perth, WA, Australia

**Keywords:** music, evolution, altruism, social cooperation, language, motherese, dopamine, oxytocin

## Abstract

Over tens of thousands of years of human genetic and cultural evolution, many types and varieties of music and language have emerged; however, the fundamental components of each of these modes of communication seem to be common to all human cultures and social groups. In this brief review, rather than focusing on the development of different musical techniques and practices over time, the main issues addressed here concern: (i) when, and speculations as to why, modern *Homo sapiens* evolved musical behaviors, (ii) the evolutionary relationship between music and language, and (iii) why humans, perhaps unique among all living species, universally continue to possess two complementary but distinct communication streams. Did music exist before language, or vice versa, or was there a common precursor that in some way separated into two distinct yet still overlapping systems when cognitively modern *H. sapiens* evolved? A number of theories put forward to explain the origin and persistent universality of music are considered, but emphasis is given, supported by recent neuroimaging, physiological, and psychological findings, to the role that music can play in promoting trust, altruistic behavior, social bonding, and cooperation within groups of culturally compatible but not necessarily genetically related humans. It is argued that, early in our history, the unique socializing and harmonizing power of music acted as an essential counterweight to the new and evolving sense of self, to an emerging sense of individuality and mortality that was linked to the development of an advanced cognitive capacity and articulate language capability.

In the twenty-first century, there are many different types and varieties of music, sung in different ways and played on many different types of musical instrument, and there are also many different types of language with different alphabets and modes of writing. Despite this biological, cultural, and behavioral diversity, the fundamental elements of music and language communication seem to be universals, present in all human cultures and social groups. There is an ever-increasing literature that focuses on the neuroscience, evolution, psychology, and sociobiology of music (e.g., Levitin, 2006; Patel, 2008; Morley, 2013; Harvey, 2017). In addressing the issue of “why music?”, a number of key questions arise, including: (i) when did our species evolve musical behaviors, (ii) what is the evolutionary relationship between music and language, and (iii) why do humans universally continue to possess two complementary but distinct communication streams, streams that are processed by some common but also some distinct pathways and circuits within the human brain.

Language is the main way we learn and pass on skills, ideas, plans, and acquired knowledge within, and across, generations. It structures thought and permits intuitive reasoning, it is expandable in content and allows the creation of virtual realities and the manipulation of symbols. But language, similar to music, also possesses emotional or prosodic content, involving changes in pitch, voice quality, loudness, and timing of delivery. Such changes convey information about the emotional state of the speaker and/or change the meaning of a word or phrase, and when vocalizing there are elements in common in the generation of phrases in music (singing) and speech (Patel, 2008). Interestingly then, studies using functional magnetic resonance imaging (fMRI) and other methods to monitor brain activity have revealed that elements of both language and music, including for example aspects of pitch and rhythm processing (Rogalsky et al., 2011; Perrachione et al., 2013), and perhaps even some elements of syntax (e.g., Sammler et al., 2013; Kunert et al., 2015, but see Bigand et al., 2014), are processed in closely related regions within the brain.

There are also, however, differences in brain circuitry. Spoken language, at least in right handers, is mostly left hemisphere biased, whereas music has a right hemisphere bias, although with training the networks involved with music processing generally show a leftward shift (Ellis et al., 2013; Thaut et al., 2014). Recent research has identified specific regions in the temporal lobe that appear to respond only to music or to speech (Angulo-Perkins et al., 2014; Norman-Haignere et al., 2015). In addition, music activates pathways and regions within the limbic system (Koelsch, 2014), a system associated with a number of functions including learning, memory, motivation, and emotional responsiveness. Music we appreciate and subjectively find arousing is associated with dopaminergic activity and activates reward centers such as the nucleus accumbens, located in the ventral striatum (Menon and Levitin, 2005; Salimpoor et al., 2011; Zatorre and Salimpoor, 2013; Mueller et al., 2015). The pathways that mediate interactions with other sensory modalities and links to motor systems controlling vocalization and body movement also show some left versus right bias for speech and music, respectively (Callan et al., 2006; Özdemir et al., 2006).

From an evolutionary perspective, the origins of modern language and articulate speech in *Homo sapiens*, and the extent to which language is encoded in the brain and/or influenced by cultural experience, remain controversial (e.g., Pinker, 1994; Hauser et al., 2002; Bickerton, 2007; Patel, 2008). Nonetheless, while obviously speculative, it seems likely that linguistic-related advances in neural hardware and software were superimposed on pre-existing neural capabilities, the latter including prosodic utterance as well as non-verbal behaviors such as hand movement (gesture), tool making, and facial expression (Mithen, 2005; Arbib et al., 2008; Aboitiz and Garcia, 2009; Fay et al., 2014). There was also likely a need for enhanced neural plasticity as well as improved attention mechanisms, greater capacity for abstract thought, a vastly improved speed of learning, and enhanced working memory and storage capability (Harvey, 2017). But if language was a key partner of our newly evolved cognitive power, and a primary driver of human cultural evolution, why did we also evolve, and why do we continue to use and enjoy, another communication stream, music? The presence of at least some shared linguistic and musical neural circuitry is not inconsistent with the proposal that, during the evolution of modern *H. sapiens*, language and music emerged from a common precursor possessed by our immediate ancestors, sometimes called a musilanguage (Brown, 2000) or protolanguage. But what does music do for us, as individuals and as a community, and where did the production and appreciation of music come from, and when? Can music have had meaningful evolutionary significance even though “it neither, plows, sows, weaves nor feeds” (Cross, 2001)?

In this brief review, the focus is on speculation about the earliest origins of music in *H. sapiens*, rather than discussing the subsequent genetic/cultural evolution of different musical genres, styles, and social practices: “Distinctions between the surface complexity of different musical styles and techniques do not tell us anything useful about the expressive purposes and power of music, or about the intellectual organization involved with its creation” (Blacking, 1976). Fossil evidence suggests that the earliest evidence of our species, *H. sapiens*, first appeared about 200,000 years ago, perhaps even as far back as 300,000 years ago (Hublin et al., 2017). Pieces of fossilized bone provide only limited information about the evolution of the modern human brain (Sherwood et al., 2008). More meaningful archeological evidence, strongly suggestive of relatively recent cognitive and behavioral advances in our species, comes from the discovery and analysis of cultural artifacts and tools (Henshilwood et al., 2002; Bouzouggar et al., 2007). Scientists can also trace lineage relationships in modern *H. sapiens*, to determine how genes, and especially the subtle regulation of those genes, have changed over the millennia. Coalescent modeling allows researchers to work backward from DNA samples from members of different human population groups in order to develop gene trees that identify the most likely common or shared ancestral population from which all living humans are derived. Lineage tracing based on changes to the Y chromosome genotype permits a study of paternal ancestry, and changes in the DNA within mitochondria allow an analysis of maternal lineage relationships (Underhill and Kivisild, 2007).

There are, of course, significant margins of error associated with such studies. Nonetheless the genetic data, when combined with evidence of cultural change, do suggest that the modern version of *H. sapiens*, the version with an advanced cognitive architecture and newly developed language capabilities, evolved within the last 100,000 years, perhaps even more recently. A study looking at the four major variants of African mitochondrial DNA has found that there was a rapid expansion of the group carrying of one of these haplotypes (L3) between 61,000 and 86,000 years ago, and that all humans living outside Africa share this phenotype (Atkinson et al., 2009; Soares et al., 2012). Within Africa, L3 carrying populations originally in Eastern Africa also increased, migrating into central and southern parts of the continent. Overall, while there were earlier migrations of *H. sapiens* out of Africa (Reyes-Centeno et al., 2014), it seems likely that the last major dispersal out of Africa by our founder population occurred somewhere between 60,000 to 70,000 years ago. Since then, human evolution has been driven by complex and dynamic interactions between genes, environment, and culture, yet both within and beyond Africa language and music remain universals, found in all living human populations and cultures. For me, the most parsimonious explanation for the universality of both communication streams is that members of our founder population possessed both systems from the very beginning (Harvey, 2017). What is clear is that by at least 43,000 years ago our ancestors were already modifying bone to make flute-like instruments (Higham et al., 2012).

Numerous theories have been put forward in an attempt to explain the seeming universality of music, from its earliest evolutionary origins through to the present day, and the possible adaptive benefits that music brought, and continues to bring, to our species (Hauser and McDermott, 2003; Huron, 2003; Mithen, 2005; Harvey, 2017). These ideas are necessarily speculative, but scientific and psychological research over the past few decades does provide some useful clues and pointers. Music and its partner dance may play an important role in mate attraction and selection, it can help structure time, and it facilitates long-distance communication. Music also enhances cognitive and motor skill development, has mnemonic power, encourages group cooperation, and social interactions, helps to define cultural identity and perhaps even facilitates conflict resolution. Of course, there may have been multiple, and intersecting, advantages for our founder population to possess musical communication as part of its make-up. Here, I wish to emphasize the early evolutionary importance of music in the context of cooperative interactions and prosocial behavior in *H. sapiens*.

One intriguing idea is that music-like capabilities may have evolved to enable mothers to interact with infants during the first few months after birth, encouraging parent-infant attachment before babies learn to talk and use language (DeCasper and Fifer, 1980; Trehub, 2000; Falk, 2004; Levitin, 2006; Trevarthan, 2008). This infant-directed communication, important in driving the behavioral, perceptual, cognitive, emotional, and social development of children, is generally known as motherese. Motherese has particular characteristics; it is rhythmic, usually delivered slowly and at higher pitch with exaggerated pitch changes that often slide from one to another, it is calming and reassuring, and is associated with other key social and emotional interactions that involve gesture, eye contact, and facial expression. However, while motherese may sound musical, the scientific evidence currently available does not, in my view, clearly establish if it is “protomusical” or “protolinguistic” in nature (Harvey, 2017). This prosodic maternal-infant communication system may in fact be more akin to the more “primitive” system used by our ancestors, prior to the more recent evolution of the two communication streams of articulate speech and music.

In adult life, speech usually involves turn taking, one person talks and another listens (Levinson, 2016), whereas music is capable of integrating and coordinating feelings and emotions within even a large group. Music, and its ally dance, require both energy and time commitment, so from an evolutionary perspective: “..if music had no value whatsoever, one might expect strong selection against musical behavior. Recent evidence from subjects with congenital amusia indicates that the necessary genetic variance is present in human populations, albeit at a very low frequency. So why have not quiet, better-rested non-musical humans out-reproduced and replaced their musical conspecifics?” (Fitch, 2006).

A possible reason for the continued universality of music, as argued in detail elsewhere (Harvey, 2017), is that the evolution of language and the modern human mind, and a new capacity for transgenerational communication and mental time travel (Suddendorf and Corballis, 2007), led to a more sharply defined sense of individuality. Our species became, as others have described it, a “society of selves” (Humphrey, 2008), with greater levels of social complexity and uncertainty, and increasingly aware of our own mortality and finite life span: “Only human beings guide their behavior by a knowledge of what happened before they were born and a preconception of what may happen after they are dead; thus only humans find their way by a light that illuminates more than the patch of ground they stand on” (Medawar, 1977). The relatively indeterminate nature of music allowed humans to interact, infer, and actively share experiences even though at the individual level each person may have been experiencing different emotions and thoughts: “Music allows participants to explore the prospective consequences of their actions and attitudes toward others within a temporal framework that promotes the alignment of participants’ sense of goals” (Cross, 2009). In other words the rewarding, harmonizing power of music and music-related activities such as dance may have acted as important and essential counterweights to the individualization experienced by increasingly intelligent, articulate, and apprehensive members of our species. As Steven Brown wrote: “…the straightforward evolutionary implication is that human musical capacity evolved because groups of musical hominids out-survived groups of non-musical hominids due to a host of factors related to group-level cooperation and coordination” (Brown, 2000).

If music did indeed play a key evolutionary role in facilitating and rewarding social bonding and mutual cooperation in *H. sapiens*, is there any evidence of this relationship at the neurological level? Neuroimaging studies have revealed that positive, optimistic thoughts activate specific regions such as the anterior cingulate cortex, the amygdala and parts of the prefrontal cortex (Sharot et al., 2007), similar to some of the regions associated with positive emotional responses to music (e.g., Salimpoor et al., 2009; Zatorre and Salimpoor, 2013; Koelsch, 2014). Altruistic acts are a form of social behavior that result in some form of benefit for the receiver and usually, but not always, at least some negative consequences for the giver. In humans, reciprocal altruism, involving mutual cooperative advantage (Trivers, 1971; Fehr and Fischbacher, 2003), has a beneficial impact not only on the viability and welfare of individuals but can also lead to better cooperation with kin as well as promote pro-social attitudes within larger groups of culturally compatible but unrelated individuals (Boyd and Richerson, 2009; Kurzban et al., 2015). Significantly, when subjects are performing mutually cooperative tasks that are rewarding and require empathy and an appreciation of social context and another person’s feelings, there is increased activity in areas such as the nucleus accumbens, amygdala, anterior cingulate cortex, superior temporal cortex, temporoparietal junction, and several specific regions in prefrontal cortex (Rilling et al., 2002, 2008; O’Doherty, 2004). Once again, these regions overlap many of the regions that are active when listening to familiar and emotionally rewarding music, music that can reinforce the sharing of affective states.

There is also neurochemical and physiological evidence to support the idea that music acts to promote empathy and cooperative behavior. Music alters the expression of a number of agents that modulate brain activity and that are associated with reward and a sense of well-being, including not only neurotransmitters such as dopamine but also several hormones (Chanda and Levitin, 2013; Zatorre and Salimpoor, 2013). Pain sensitivity, thought to be a surrogate for altered endogenous opioid levels, is lowered during participation in group music activities, including choral singing and dance (Dunbar et al., 2012; Tarr et al., 2015), and music can reduce levels of the stress hormone cortisol (Khalfa et al., 2003).

In humans the hormone oxytocin has been shown to promote the expression of positive emotions and prosocial altruistic attitudes (Hurlemann et al., 2010; Marsh et al., 2015; Leppanen et al., 2017). When applied as a spray via the nose, oxytocin reduces anxiety and fosters trust and emotional empathy (Kosfeld et al., 2005; Baumgartner et al., 2008), and genetic variants of the oxytocin receptor are linked to differences in the way humans empathize and engage in prosocial activities (Rodrigues et al., 2009; Aspé-Sánchez et al., 2016). In a study that also analyzed stress and cortisol levels in participants, it was recently reported that oxytocin levels measured in saliva are *decreased* during choral but not solo singing (Schladt et al., 2017). However, measurement of salivary oxytocin levels do not necessarily reflect levels within the specific regions of the brain that possess oxytocin receptors (Mitre et al., 2016; Lin et al., 2018), and there is a clearer and more consistent relationship between blood plasma levels of oxytocin and levels found in cerebrospinal fluid (Valstad et al., 2017). Interestingly then, circulating levels of the hormone oxytocin were found to be increased only under conditions in which singers were asked to *improvise* together (Keeler et al., 2015). Musical improvisation, presumably comparable to the type of activity experienced by early humans, requires that participants innovate, engage, and socially interact with other members of the group. Consistent with this, compared to other creative group activities, singing encourages more rapid inter-individual bonding, apparently short-circuiting the usual way that social relationships are established: “The capacity of singing to bond groups of relative strangers in humans may have played a crucial role in allowing modern humans to create and maintain much larger social networks than their evolutionary relatives…” (Pearce et al., 2015).

For many thousands of years, the evolution and development of musical styles and practices, as well as individual musical abilities, has been driven by a complex interplay between genes and culture. Evidence for the vertical transmission of at least some aspects of musical behavior in human populations comes from studies showing that genetic variants in a number of neuromodulator receptors are linked to musical memory, aptitude, and creativity (Liu et al., 2016; Oikkonen et al., 2016; Mariath et al., 2017). Furthermore, lineage studies have revealed a correlation between variation in folk musical styles and genetic variance within populations (Pamjav et al., 2012; Brown et al., 2013; Le Bomin et al., 2016), all lending support to the proposal that musical traits are heritable.

In summary, it is argued that music and music-related behaviors, operating alongside language and articulate speech, were important in helping to promote emotional synergy, social bonding, and foster group-level cooperation and coordination early in human evolution. As suggested elsewhere (Cross, 2003), music may have been especially suited for this purpose because it is usually risk free and of indeterminate meaning. In a group context, music allows humans to interact and share experiences during a particular musical activity even though each person may have very different outlooks and goals, and be involved in a range of real or imagined relationships. “Wherever humans live, and however, they have organized their societies, they exhibit a behavioral peculiarity of gathering from time to time to sing and dance together in a group. By featuring both human song and entrainment (in the dancing movements and perhaps clapping performed in synchrony with the singing/music), such behavior qualifies as human music. Indeed, the fact that it occurs in every human culture, and indeed subculture, without exception, unless deliberately suppressed by severe sanctions against it, marks this phenomenon as the most universal human behavior of a musical kind on record” (Merker et al., 2015).

An appreciation of the evolutionary significance of music, and the impact that the universal of music has had in promoting social harmony and an individual’s sense of well-being in our species, can only help when advocating for the importance of music in education and for therapeutic purposes in the clinic (Harvey, 2012). Music and associated synchronized behaviors remain core human attributes, promoters of prosocial, empathic behaviors, and cultural cohesion, and it is to be hoped that humans will continue to harness music’s power, for the good of society.

## Author Contributions

The author confirms being the sole contributor of this work and approved it for publication.

## Conflict of Interest Statement

The author declares that the research was conducted in the absence of any commercial or financial relationships that could be construed as a potential conflict of interest.

## References

[B1] Aboitiz F. Garcia R. (2009). Merging of phonological and gestural circuits in early language evolution. 20 71–84. 10.1515/REVNEURO.2009.20.1.71 19526735

[B2] Angulo-Perkins A. Aubé W. Peretz I. Barios F. A. Armony J. L. Concha L. (2014). Music listening engages specific cortical regions within the temporal lobes: differences between musicians and non-musicians. 59 126–137. 10.1016/j.cortex.2014.07.013 25173956

[B3] Arbib M. A. Liebal K. Pika S. (2008). Primate vocalization, gesture, and the evolution of human language. 49 1053–1063. 10.1086/593015 19391445

[B4] Aspé-Sánchez M. Moreno M. Rivera M. I. Rossi A. Ewer J. (2016). Oxytocin and vasopressin receptor gene polymorphisms: role in social and psychiatric traits. 9:510 10.3389/fnins.2015.00510 PMC4729929 26858594

[B5] Atkinson Q. D. Gray R. D. Drummond A. J. (2009). Bayesian coalescent interference of major human mitochondrial DNA haplotype expansions in Africa. 276 367–373. 10.1098/rspb.2008.0785 18826938 PMC2674340

[B6] Baumgartner T. Heinrichs M. Vonlanthen A. Fischbacher U. Fehr E. (2008). Oxytocin shapes the neural circuitry of trust and adaptation in humans. 58 639–650. 10.1016/j.neuron.2008.04.009 18498743

[B7] Bickerton D. (2007). Language evolution: a brief guide for linguists. 117 510–526. 10.1016/j.lingua.2005.02.006

[B8] Bigand E. Delbé C. Poulin-Charronnat B. Leman M. Tillmann B. (2014). Empirical evidence for musical syntax processing? Computer simulations revel the contribution of auditory short-term memory. 8:94. 10.3389/fnsys.2014.00094 24936174 PMC4047967

[B9] Blacking J. (1976). London: Faber and Faber.

[B10] Bouzouggar A. Barton N. Vanhaeren M. d’Errico F. Collcutt S. Higham T. (2007). 82,000-year-old shell beads from North Africa and implications for the origins of modern human behavior. 104 9964–9969. 10.1073/pnas.0703877104 17548808 PMC1891266

[B11] Boyd R. Richerson P. J. (2009). Culture and the evolution of human cooperation. 364 3281–3288. 10.1098/rstb.2009.0134 19805434 PMC2781880

[B12] Brown S. (2000). “The “musilanguage” model of human evolution,” in , eds Wallin N. Merker B. Brown S. (Cambridge MA: MIT Press), 271–300.

[B13] Brown S. Savage P. E. Ko A. M. Stoneking M. Ko Y. C. Loo J. H. (2013). Correlations in the population structure of music, genes and language. 281:20132072. 10.1098/rspb.2013.2072 24225453 PMC3843827

[B14] Callan D. E. Tsytsarev V. Hanakawa T. Callan A. M. Katsuhara M. Fukuyama H. (2006). Song and speech: brain regions involved with perception and covert production. 31 1327–1342. 10.1016/j.neuroimage.2006.01.036 16546406

[B15] Chanda M. L. Levitin D. J. (2013). The neurochemistry of music. 17 179–193. 10.1016/j.tics.2013.02.007 23541122

[B16] Cross I. (2001). Music, mind and evolution. 29 95–112. 10.1177/0305735601291007

[B17] Cross I. (2003). “Music, cognition, culture and evolution,” in , eds Peretz I. Zatorre R. (Oxford: Oxford University Press), 42–56. 10.1093/acprof:oso/9780198525202.003.0004

[B18] Cross I. (2009). The evolutionary nature of musical meaning. 179–200. 10.3389/fpsyg.2017.00494 28421015 PMC5378764

[B19] DeCasper A. J. Fifer W. P. (1980). Of human bonding: newborns prefer their motheers’ voices. 208 1174–1176. 10.1126/science.7375928 7375928

[B20] Dunbar R. I. M. Kaskatis K. MacDonald I. Barra V. (2012). Performance of music elevates pain threshold and positive affect: implications for the evolutionary function of music. 10 688–702. 10.1177/147470491201000403 23089077 PMC10426978

[B21] Ellis R. J. Bruijn B. Norton A. C. Winner E. Schlaug G. (2013). Training-mediated leftward asymmetries during music processing: a cross-sectional and longitudinal fMRI analysis. 75 97–107. 10.1016/j.neuroimage.2013.02.045 23470982 PMC3705762

[B22] Falk D. (2004). Prelinguistic evolution in early hominids: whence motherese? 27 491–503. 15773427 10.1017/s0140525x04000111

[B23] Fay N. Lister C. J. Ellison T. M. Goldin-Meadow S. (2014). Creating a communication system from scratch: gesture beats vocalization hands down. 5:354. 10.3389/fpsyg.2014.00354 24808874 PMC4010783

[B24] Fehr E. Fischbacher U. (2003). The nature of human altruism. 425 785–791. 10.1038/nature02043 14574401

[B25] Fitch T. W. (2006). The biology and evolution of music: a comparative perspective. 100 173–215. 10.1016/j.cognition.2005.11.009 16412411

[B26] Harvey A. R. (2012). “Evolution, music and neurotherapy,” in , ed. Poiani A. (Cambridge: Cambridge University Press), 150–163.

[B27] Harvey A. R. (2017). Oxford: Oxford University Press 10.1093/acprof:oso/9780198786856.001.0001

[B28] Hauser M. D. Chomsky N. Fitch W. T. (2002). The faculty of language: what is it, who has it, and how did it evolve? 298 1569–1579. 10.1126/science.298.5598.1569 12446899

[B29] Hauser M. D. McDermott J. (2003). The evolution of the music faculty: a comparative perspective. 6 663–668. 10.1038/nn1080 12830156

[B30] Henshilwood C. S. d’Errico F. Yates R. Jacobs Z. Tribolo C. Duller G. A. (2002). Emergence of modern human behaviour: Middle Stone Age engravings from South Africa. 295 1278–1280. 10.1126/science.1067575 11786608

[B31] Higham T. Basell L. Jacobi R. Wood R. Bronk Ramsey C. Conard N. J. (2012). Testing models for the beginnings of the Aurignacian and the advent of figurative art and music: the radiocarbon chronology of Geißenklösterle. 62 664–676. 10.1016/j.jhevol.2012.03.003 22575323

[B32] Hublin J. J. Ben-Ncer A. Bailey S. E. Freidline S. E. Neubauer S. Skinner M. M. (2017). New fossils from Jebel Irhoud, Morocco and the pan-African origin of Homo sapiens. 546 293–296. 10.1038/nature22335 28593953

[B33] Humphrey N. (2008). The society of selves. 362 745–754. 10.1098/rstb.2006.2007 17255004 PMC2346528

[B34] Hurlemann R. Patin A. Onur O. A. Cohen M. X. Baumgartner T. Metzler S. (2010). Oxytocin enhances amygdala-dependent, socially reinforced learning and emotional empathy in humans. 30 4999–5007. 10.1523/JNEUROSCI.5538-09.2010 20371820 PMC6632777

[B35] Huron D. (2003). “Is music an evolutionary adaptation?,” in , eds Peretz I. Zatorre R. L. (Oxford: Oxford University Press).

[B36] Keeler J. R. Roth E. A. Neuser B. L. Spitsbergen J. M. Waters D. J. Vianney J. M. (2015). The neurochemistry and social flow of singing: bonding and oxytocin. 9:518. 10.3389/fnhum.2015.00518 26441614 PMC4585277

[B37] Khalfa S. Bella S. D. Roy M. Peretz I. Lupien S. J. (2003). Effects of relaxing music on salivary cortisol level after psychological stress. 999 374–376. 10.1196/annals.1284.045 14681158

[B38] Koelsch S. (2014). Brain correlates of music-evoked emotions. 15 170–180. 10.1038/nrn3666 24552785

[B39] Kosfeld M. Heinrichs M. Zak P. J. Fischbacher U. Fehr E. (2005). Oxytocin increases trust in humans. 435 673–676. 10.1038/nature03701 15931222

[B40] Kunert R. Willems R. M. Casasanto D. Patel A. D. Hagoort P. (2015). Music and language syntax interact in Broca’s area: an fMRI study. 10:e0141069. 10.1371/journal.pone.0141069 26536026 PMC4633113

[B41] Kurzban R. Burton-Chellew M. N. West S. A. (2015). The evolution of altruism in humans. 66 575–599. 10.1146/annurev-psych-010814-015355 25061670

[B42] Le Bomin S. Lecointre G. Heyer E. (2016). The evolution of musical diversity: the key role of vertical transmission. 11:e0151570. 10.1371/journal.pone.0151570 27027305 PMC4814106

[B43] Leppanen J. Ng K. W. Tchanturia K. Treasure J. (2017). Meta-analysis of the effects of intranasal oxytocin on interpretation and expression of emotions. 78 125–144. 10.1016/j.neubiorev.2017.04.010 28467893

[B44] Levinson S. C. (2016). Turn-taking in human communication – origins and implications for language processing. 20 6–14. 10.1016/j.tics.2015.10.010 26651245

[B45] Levitin D. J. (2006). New York, NY: Dutton.

[B46] Lin Y.-T. Hsieh T.-Y. Tsai T.-C. Chen C.-C. Huang C.-C. Hsu K.-S. (2018). Conditional deletion of hippocampal CA2/CA3a oxytocin receptors impairs the persistence of long-term social recognition memory in mice. 38 1218–1231. 10.1523/JNEUROSCI.1896-17.2017 29279308 PMC6596267

[B47] Liu X. Kanduri C. Oikkonen J. Karma K. Raijas P. Ukkola-Vuoti L. (2016). Detecting signatures of positive selection associated with musical aptitude in the human genome. 6:21198. 10.1038/srep21198 26879527 PMC4754774

[B48] Mariath L. M. Silva A. M. D. Kowalski T. W. Gattino G. S. Araujo G. A. Figueiredo F. G. (2017). Music genetics research: association with musicality of a polymorphism in the AVPRA gene. 40 421–429. 10.1590/1678-4685-GMB-2016-0021 28534928 PMC5488451

[B49] Marsh N. Scheele D. Gerhardt H. Strang S. Enax L. Weber B. (2015). The neuropeptide oxytocin induces a social altruism bias. 35 15696–15701. 10.1523/JNEUROSCI.3199-15.2015 26609161 PMC6705471

[B50] Medawar P. B. (1977). London: Wildwood House.

[B51] Menon V. Levitin D. J. (2005). The rewards of music listening: response and physiological connectivity of the mesolimbic system. 28 175–184. 10.1016/j.neuroimage.2005.05.053 16023376

[B52] Merker B. Morley I. Zuidema W. (2015). Five fundamental constraints on theories of the origins of music. 370:20140095. 10.1098/rstb.2014.0095 25646518 PMC4321136

[B53] Mithen S. (2005). London: Orion.

[B54] Mitre M. Marlin B. J. Schiavo J. K. Morina E. Norden S. E. Hackett T. A. (2016). A distributed network for social cognition enriched for oxytocin receptors. 36 2517–2535. 10.1523/JNEUROSCI.2409-15.2016 26911697 PMC4764667

[B55] Morley I. (2013). Oxford: Oxford University Press 10.1093/acprof:osobl/9780199234080.001.0001

[B56] Mueller K. Fritz T. Mildner T. Richter M. Schulze K. Lepsien J. (2015). Investigating the dynamics of the brain response to music: a central role of the ventral striatum/nucleus accumbens. 116 68–79. 10.1016/j.neuroimage.2015.05.006 25976924

[B57] Norman-Haignere S. Kanwisher N. G. McDermott J. H. (2015). Distinct cortical pathways for music and speech revealed by hypothesis-free voxel decomposition. 88 1281–1296. 10.1016/j.neuron.2015.11.035 26687225 PMC4740977

[B58] O’Doherty J. P. (2004). Reward representations and reward-related learning in the human brain: insights from neuroimaging. 14 769–776. 10.1016/j.conb.2004.10.016 15582382

[B59] Oikkonen J. Onkamo P. Järvelä I. Kanduri C. (2016). Convergent evidence for the molecular basis of musical traits. 6:39707. 10.1038/srep39707 28004803 PMC5177873

[B60] Özdemir E. Norton A. Schlaug G. (2006). Shared and distinct neural correlates of singing and speaking. 33 628–635. 10.1016/j.neuroimage.2006.07.013 16956772

[B61] Pamjav H. Juhász Z. Zalán A. Németh E. Damdin B. (2012). A comparative phylogenetic study of genetics and folk music. 287 337–349. 10.1007/s00438-012-0683-y 22392540

[B62] Patel A. D. (2008). New York: Oxford University Press.

[B63] Pearce E. Launay J. Dunbar R. I. M. (2015). The ice-breaker effect: singing mediates fast social bonding. 2:150221. 10.1098/rsos.150221 26587241 PMC4632513

[B64] Perrachione T. K. Fedorenko E. G. Vinke L. Gibson E. Dilley L. C. (2013). Evidence for shared cognitive processing of pitch in music and language. 8:e73372. 10.1371/journal.pone.0073372 23977386 PMC3744486

[B65] Pinker S. (1994). London: Penguin 10.1037/e412952005-009

[B66] Reyes-Centeno H. Ghirotto S. Détroit F. Grimaud-Hervé D. Barbujani A. G. Harvati K. (2014). Genomic and cranial phenotype data support multiple modern human dispersals from Africa and a southern route into Asia. 111 7248–7253. 10.1073/pnas.1323666111 24753576 PMC4034217

[B67] Rilling J. K. Gutman D. Zeh T. Pagnoni G. Berns G. Kilts C. (2002). A neural basis for social cooperation. 67 511–521. 10.1016/S0896-6273(02)00755-9 12160756

[B68] Rilling J. K. King-Cass B. Sanfey A. G. (2008). The neurobiology of social decision-making. 18 159–165. 10.1016/j.conb.2008.06.003 18639633

[B69] Rodrigues S. M. Saslow L. R. Garcia N. John O. P. Keltner D. (2009). Oxytocin receptor genetic variation relates to empathy and stress reactivity in humans. 106 21437–21441. 10.1073/pnas.0909579106 19934046 PMC2795557

[B70] Rogalsky C. Rong F. Saberi K. Hickok G. (2011). Functional anatomy of language and music perception: temporal and structural factors investigated using functional magnetic resonance imaging. 31 3843–3852. 10.1523/JNEUROSCI.4515-10.2011 21389239 PMC3066175

[B71] Salimpoor V. N. Benovoy M. Larcher K. Dagher A. Zatorre R. J. (2011). Anatomically distinct dopamine release during anticipation and experience of peak emotion in music. 14 257–264. 10.1038/nn.2726 21217764

[B72] Salimpoor V. N. Benovoy M. Longo G. Cooperstock J. R. Zatorre R. J. (2009). The rewarding aspects of music listening are related to the degree of emotional arousal. 4:e7487. 10.1371/journal.pone.0007487 19834599 PMC2759002

[B73] Sammler D. Koelsch S. Ball T. Brandt A. Grigutsch M. Huppertz H. J. (2013). Co-localizing linguistic and musical syntax with intracranial EEG. 64 134–146. 10.1016/j.neuroimage.2012.09.035 23000255

[B74] Schladt T. M. Nordmann G. C. Emilius R. Kudielka B. M. de Jong T. R. Neumann I. D. (2017). Choir versus solo singing: effects on mood, and salivary oxytocin and cortisol concentrations. 11:430. 10.3389/fnhum.2017.00430 28959197 PMC5603757

[B75] Sharot T. Riccardi A. M. Raio C. M. Phelps E. A. (2007). Neural mechanisms mediating optimism bias. 450 102–105. 10.1038/nature06280 17960136

[B76] Sherwood C. C. Subiaul F. Zawidzki T. W. (2008). A natural history of the human mind: tracing evolutionary changes in brain and cognition. 212 426–454. 10.1111/j.1469-7580.2008.00868.x 18380864 PMC2409100

[B77] Soares P. Alshamali F. Pereira J. B. Fernandes V. Silva N. M. Afonso C. (2012). The expansion of mtDNA haplogroup L3 within and out of Africa. 29 915–927. 10.1093/molbev/msr245 22096215

[B78] Suddendorf Y. Corballis M. C. (2007). The evolution of foresight: what is mental time travel, and is it unique to humans? 30 299–351. 10.1017/S0140525X07001975 17963565

[B79] Tarr B. Launay J. Cohen E. Dunbar R. (2015). Synchrony and exertion during dance independently raise pain threshold and encourage social bonding. 11:20150767. 10.1098/rsbl.2015.0767 26510676 PMC4650190

[B80] Thaut M. H. Trimarchi P. D. Parsons L. M. (2014). Human brain basis of musical rhythm perception: common and distinct neural substrates for meter, tempo, and pattern. 4 428–452. 10.3390/brainsci4020428 24961770 PMC4101486

[B81] Trehub S. (2000). “Human processing dispositions and musical universals,” in , eds Wallin N. L. Merker B. Brown S. (Cambridge, MA: MIT Press), 427–448.

[B82] Trevarthan C. (2008). The musical art of infant conversation: narrating in the time of sympathetic experience, without rational interpretation, before words. 12(Suppl.), 15–46. 10.1177/1029864908012001021

[B83] Trivers R. L. (1971). The evolution of reciprocal altruism. 46 35–57. 10.1086/406755

[B84] Underhill P. A. Kivisild T. (2007). Use of Y chromosome and mitochondrial DNA population structure in tracing human migrations. 41 539–564. 10.1146/annurev.genet.41.110306.130407 18076332

[B85] Valstad M. Alvares G. A. Egknud M. Matziorinis A. M. Andreassen O. A. Westlye L. T. (2017). The correlation between central and peripheral oxytocin concentrations: a systematic review and meta-analysis. 78 117–124. 10.1016/j.neubiorev.2017.04.017 28442403

[B86] Zatorre R. J. Salimpoor V. N. (2013). From perception to pleasure: music and its neural substrates. 110 10430–10437. 10.1073/pnas.1301228110 23754373 PMC3690607

